# Generation and Characterization of the Western Regional Research Center Brachypodium T-DNA Insertional Mutant Collection

**DOI:** 10.1371/journal.pone.0041916

**Published:** 2012-09-17

**Authors:** Jennifer N. Bragg, Jiajie Wu, Sean P. Gordon, Mara E. Guttman, Roger Thilmony, Gerard R. Lazo, Yong Q. Gu, John P. Vogel

**Affiliations:** 1 United States Department of Agriculture- Agriculture Research Service (USDA-ARS), Western Regional Research Center, Albany, California, United States of America; 2 University of California Davis, Davis, California, United States of America; University of Massachusetts Amherst, United States of America

## Abstract

The model grass *Brachypodium distachyon* (*Brachypodium*) is an excellent system for studying the basic biology underlying traits relevant to the use of grasses as food, forage and energy crops. To add to the growing collection of *Brachypodium* resources available to plant scientists, we further optimized our *Agrobacterium tumefaciens*-mediated high-efficiency transformation method and generated 8,491 *Brachypodium* T-DNA lines. We used inverse PCR to sequence the DNA flanking the insertion sites in the mutants. Using these flanking sequence tags (FSTs) we were able to assign 7,389 FSTs from 4,402 T-DNA mutants to 5,285 specific insertion sites (ISs) in the *Brachypodium* genome. More than 29% of the assigned ISs are supported by multiple FSTs. T-DNA insertions span the entire genome with an average of 19.3 insertions/Mb. The distribution of T-DNA insertions is non-uniform with a larger number of insertions at the distal ends compared to the centromeric regions of the chromosomes. Insertions are correlated with genic regions, but are biased toward UTRs and non-coding regions within 1 kb of genes over exons and intron regions. More than 1,300 unique genes have been tagged in this population. Information about the Western Regional Research Center *Brachypodium* insertional mutant population is available on a searchable website (http://brachypodium.pw.usda.gov) designed to provide researchers with a means to order T-DNA lines with mutations in genes of interest.

## Introduction

*Brachypodium distachyon* (*Brachypodium*) is an annual grass native to the Mediterranean and Middle East and is a member of the Pooideae subfamily [1]. This group also contains cereal and forage grasses including economically important species with complex genomes such as *Triticum aestivum* (bread wheat), *Hordeum vulgare* (barley), and *Avena sativa* (oats). In 2001, *Brachypodium* was proposed as a model system for the study of the Triticeae [2], and in 2005 following a feasibility study from the United States Departments of Energy on the use of plant biomass for the generation of energy and other products, *Brachypodium* was recognized as an attractive model for the improvement of proposed bioenergy feedstocks such as switchgrass and *Miscanthus* [3]. Many aspects of grass biology, such as cell wall composition and architecture [4], [5], development, and grain properties, are distinct from dicots. In these cases, a grass such as *Brachypodium*, that possesses all of the biological, physical and genomic attributes required for an experimental system [2], [6], [7], represents a more relevant model than the dicot model *Arabidopsis*. *Brachypodium's* compact 272 Mbp diploid genome is similar to rice and sorghum in gene content and gene family structure [8], and the small size, rapid generation time, and simple growth requirements of *Brachypodium* enable high-throughput studies that are not feasible using larger, more demanding species [2], [9]–[11]. Additionally, *Brachypodium* is self-fertile and rarely outcrosses [12] which facilitates breeding homozygous lines for applications that require the maintenance of large numbers of independent genotypes, such as mapping experiments, mutant analysis, and studies of natural diversity [13]–[17].

Over the last decade, several groups have developed resources that allow *Brachypodium* to serve as powerful functional and structural genomic model system. These resources include highly efficient transformation [18], [19] and crossing protocols (http://brachypodium.pw.usda.gov/), bacterial artificial chromosome (BAC) libraries [20]–[22], genetic markers [12], [23], genetic linkage and physical maps [24]–[26], and a continually increasing germplasm collection [12], [27], [28]. Publicly accessible databases provide access to the complete genome sequence of *Brachypodium* accession Bd21 [8], array expression data (http://www.brachypodium.org/), and comparative genomics tools (http://brachypodium.pw.usda.gov, http://www.brachybase.org, http://www.phytozome.net, http://www.modelcrop.org, http://mips.helmholtz-muenchen.de/plant/). The surge of publications in the year following the release of the complete genome sequence testifies to the utility of these *Brachypodium* resources for the study of cereal, forage, and bioenergy grasses in areas as diverse as grass flowering time [29]–[31], drought response [29], seed dormancy [32], grain development [33], [34], iron homeostasis [35], transcription factors [30], [36], cell wall composition [37], disease resistance [38], complex genome sequencing [39], and genome evolution [40] (for a review see [9]). Forthcoming sequencing data for more than 50 additional accessions and recombinant inbred lines will serve to increase the utility of *Brachypodium* as an experimental system for the grasses.

While comparative analyses of sequence information can provide educated guesses about gene function, establishing a true link between genes and their biological function requires detailed functional characterization. Sequence indexed insertional mutants are a particularly valuable tool in this context because mutations in a given gene can be identified by simply searching a database. Thus, the large, sequence-indexed T-DNA and transposon-tagged mutant collections available for Arabidopsis and rice are an invaluable resource for such forward genetic studies. In addition to providing loss of function mutants when a T-DNA or transposon lands in a gene, the vectors can be designed to track promoter function and overexpress nearby genes. Gene trapping vectors containing promoterless reporter genes with splice donor and acceptor sites can be used to infer the expression pattern of disrupted genes and to identify promoters with tissue-specific expression patterns [41], [42]. Activation tagging vectors contain transcriptional enhancers that cause nearby genes to be overexpressed while maintaining normal expression patterns. Such activation tagged mutants are particularly useful for investigating essential genes in which disruption is lethal, genes with redundant functions where a knockout in one family member fails to produce a phenotype, and the regulation of complex processes in which the activation of a global control gene is required to observe a phenotype [43]–[45].

A high efficiency transformation method is a prerequisite for the creation of large T-DNA collections. Fortunately, *Brachypodium* is highly amenable to tissue culture and transformation. After conditions were established for the generation of embryogenic callus and production and regeneration of fertile *Brachypodium* plants [46], *Brachypodium* was successfully transformed using a biolistic method [2], [47]. Due to complex insertion patterns that often involve many copies of the inserted DNA and local rearrangements, biolistic transformation is not ideal for the generation of mutant populations. *Agrobacterium*-mediated transformation of *Brachypodium* was first reported in 2006 [27]. Subsequently, two groups optimized high efficiency *Agrobacterium*-mediated transformation methods [18], [19]. With efficient transformation methods in hand, the generation of *Brachypodium* T-DNA collections was initiated. In 2010, the BrachyTAG collection (http://www.brachytag.org) [48] reported 741 T-DNA lines with flanking sequence tags (FSTs) anchoring insertions sites within the *Brachypodium* genome. These lines contain insertions between 1,500 bp upstream to 500 bp downstream of the coding sequence for 364 *Brachypodium* genes. Based on this data, the authors estimated that at least 51,976 lines would be necessary to obtain a T-DNA insertion in any gene with a 95% probability and that the actual number of lines required to meet this goal is over 100,000. As a means to approach this distant target, we developed a high-throughput pipeline for the production and sequencing of *Brachypodium* T-DNA mutants. Here we present the optimization of this pipeline including a comparison of T-DNA and transposon tagging strategies, further optimization of our transformation method, and a comparison of several T-DNA vectors. We used these optimized methods to generate 8,491 *Brachypodium* T-DNA mutants and identify 5,285 unique insertion loci for 4,402 of these lines.

## Materials and Methods

### Plant lines and growth conditions

*Brachypodium* inbred lines Bd21 and Bd21-3 were compared in initial transformation studies, and Bd21-3 was selected to generate the bulk of the T-DNA mutant population [18]. Plants were grown in a soil mix of 1 part sandy loam, 2 parts sand, 3 parts peat moss, and 3 parts medium grade (#3) vermiculite. A time release fertilizer containing micronutrients (Osmocote Plus 15-9-12, Scotts Co., Marysville, OH) was added at the time of planting. Plants were grown in both greenhouses and growth chambers. Growth chambers conditions were 20 hr light ∶ 4 hr dark photoperiod, cool-white fluorescent lighting at a level of 150 µEm^−2^ s^−1^, and temperatures of 24°C during the day and 18°C at night. Greenhouse conditions were no shading, 24°C in the day and 18°C at night with the day length extended to 16 hours by supplemental lighting. T_1_ seeds were harvested from senesced T_0_ plants after they were completely dried (typical yield ranged between 50–150 T_1_ seeds per plant). When additional seeds were required for particular lines, 6–12 T_1_ seeds were sown and the harvested seeds were collected in bulk. If large quantities of seeds are required, plants can be grown under short day conditions and then vernalized or moved to long day conditions. In this case, over 1,000 seeds can be obtained from an individual plant.

### T-DNA constructs

**Figure 1:**
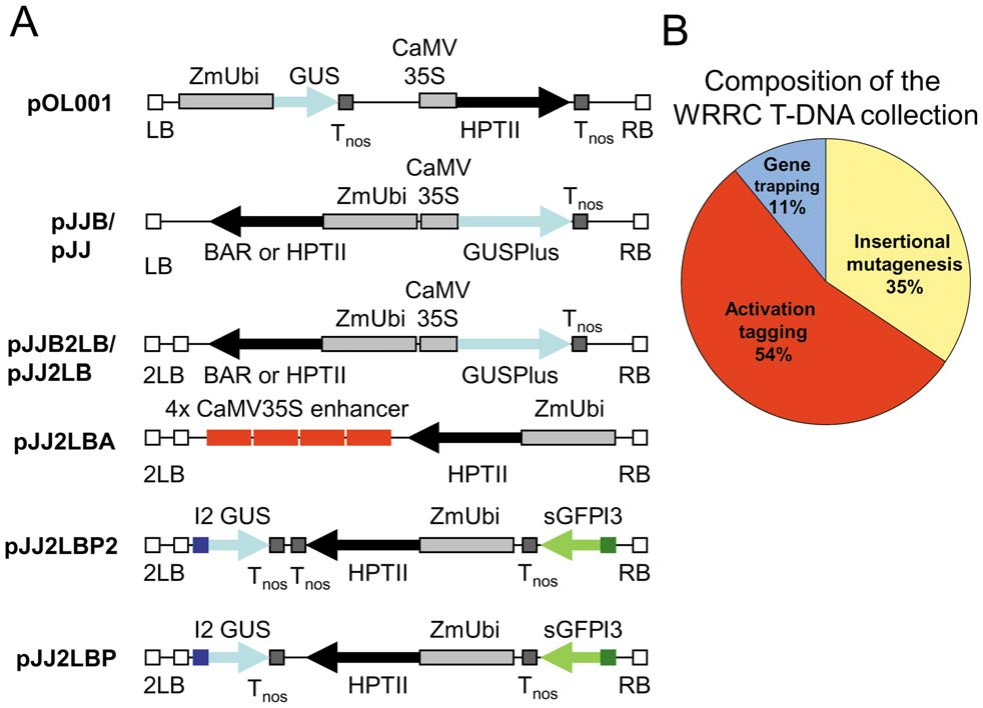
T-DNA constructs used to generate the USDA-ARS-WRRC T-DNA collection. **A.** Illustrations of the T-DNA regions of pOL001 and the seven new constructs used to generate the majority of the population are shown. White boxes: T-DNA left border (LB or 2LB) and right border sequences (RB). Light gray boxes: *Z. mays* ubiquitin promoter with intron (ZmUbi) and cauliflower mosaic virus 35S promoter (CaMV 35S). Dark grey boxes: nos terminator sequences (T_nos_). Red boxes: cauliflower mosaic virus 35S transcriptional enhancer sequences (4× CaMV35S enhancer). Black arrows: hygromycin phosphotransferase selection gene (*HptII*) or phosphinothricin acetyltransferase selection gene (*BAR*). Blue arrows and boxes: ß-glucuronidase reporter genes (*GUS* and *GUSPlus*) with a rice tubulin intron containing splice donor and acceptor sites (I2). Green arrow and box: green fluorescent protein reporter gene (*sGFP*) with a rice tubulin intron containing splice donor and acceptor sites (I3). **B.** Pie chart showing the composition of the T-DNA population. The yellow represents lines made with the insertional mutagenesis vectors including pOL001, pJJH, pJJB, pJJ2LB, and pJJB2LB. The blue represents lines made with the gene trap vectors pJJ2LBP and pJJ2LBP2, and the red represents lines made with the activation tagging vector pJJ2LBA.

Several constructs were used in this study. The previously described construct pOL001 [27] was used as a benchmark for the evaluation of the new constructs and for the initial production of T-DNA lines. pOL001 contains a *HptII* gene under control of the CaMV 35S promoter for selection of transgenic callus and a GUS reporter gene under control of the maize ubiquitin promoter (**Fig. 1A**).

**Figure 2:**
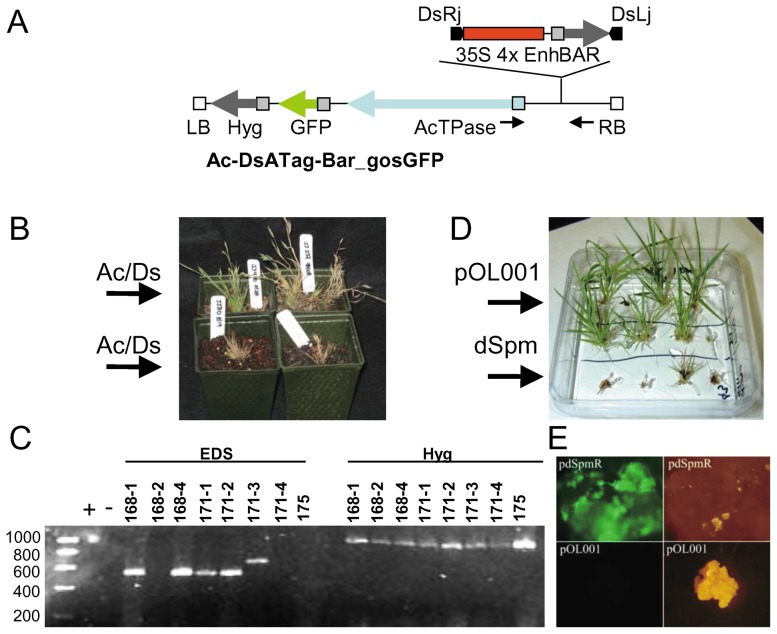
Evaluation of transposon tagging in *Brachypodium*. **A.** Diagram of the T-DNA region of Ac-DsATag-Bar_gosGFP. The mobile element is depicted on the upper line. Note that when the mobile element excises the empty donor site (EDS) can be amplified by the flanking PCR primers, solid black arrows. **B.** Examples of transgenic plants created using Ac-DsATag-Bar_gosGFP. Most plants containing the transposable elements died before flowering or failed to set seed. **C.** PCR using primers that flank the Ds mobile element. An EDS produces a band while an intact donor site will not produce a band. Note that five of eight lines contain an EDS indicating that Ds was excised. The hygromycin resistance gene was amplified from all lines indicating that they contained at least part of the T-DNA. **D.** Examples of transgenic plants created using the pdSpm-R tagging construct and the control vector pOL001. Most plants containing the transposable elements died when very small. **E.** Calluses transformed with the pdSpm-R tagging construct were fluorescent green due to the expression of GFP contained on the T-DNA. By contrast, calluses transformed with a control plasmid, pOL001, were not fluorescent. For both constructs, pictures on the left are taken under UV light through a GFP filter and the pictures on the right are under white light.

Ac-Ds and En-I(Spm) transposon systems were tested using the constructs Ac-DsATag-Bar_gosGFP [49] (**Fig. 2A**) and pdSpm-R [50]. These constructs are single vector transposon tagging constructs and contain both the transposase and the corresponding mobile element. We built seven additional constructs (the pJJ vectors) starting from pCAMBIA vector backbones (http://www.cambia.org/) (**Fig. 1A**). The first constructs (pJJ, pJJB) were used to compare the transformation efficiency of hygromycin and BASTA selections. The pJJ2LB and pJJB2LB constructs were used to determine if the presence of two left border sequences improved the efficiency of FST generation by decreasing the transfer of vector DNA beyond the left border [51]. The final vectors were modified from pJJ2LB to create derivatives designed for gene trapping (pJJ2LBP and pJJ2LBP2) and activation tagging (pJJ2LBA). The construction of these vectors is described below, and primers used in their construction are listed in **Table S1**. Sequencing was performed on the T-DNA regions of all constructs to ensure that no mutations were introduced during PCR and to confirm proper orientation after ligation of digestion fragments.

The pUbi-BASK vector (courtesy of Jim Thomson) was the source of the maize ubiquitin promoter with the ubiquitin 5′ intron used in the constructs below. To generate pUbi-BASK the pAHC20 vector [52] had the internal EcoRI restriction site within the maize ubiquitin promoter removed using site directed mutagenesis. The *BAR* coding sequence was excised from pAHC20 with BamHI and KpnI and a BamHI/AscI/SpeI/KpnI (BASK) multiple cloning site was inserted in its place.

#### pJJB

This vector has a phosphinothricin acetyl transferase selection (*BAR*) gene under the control of the maize ubiquitin promoter. The *BAR* gene sequence was amplified from pMNRTT224nb (courtesy of Venkatesan Sundaresan) using the BARBamHIF and BARKpnIR primers. The PCR product was digested with BamHI and KpnI and inserted into the corresponding sites of the pUbi-BASK vector directly after the maize ubiquitin promoter with the ubiquitin 5′ intron and preceding the nos terminator. The Ubi-BAR-nos fragment was then inserted into the HindIII and EcoRI sites of the pCAMBIA0305.2 vector (http://www.cambia.org/). The resulting construct also has a GUSPlus reporter gene under control of the CaMV 35S promoter and a kanamycin resistance gene for selection in bacteria.

#### pJJB2LB

To generate this vector the pCAMBIA0305.2 left border (LB) was replaced with the double left border sequence (2LB) from pL3 [53]. This 741 base pair region consists of a nopaline LB and its flanking regions followed by an octopine LB and its flanking regions. The 2LB sequence was amplified with primers designed to introduce a SacII site adjacent to the octopine border and an AseI site adjacent to the nopaline border (pL3-2LB-F1 and pL3-2LB-R1). The 2LB PCR fragment was introduced between the AseI and SacII sites of pCAMBIA0305.2. Then, the Ubi-BAR-nos sequence (as for pJJB) was introduced between the HindIII and EcoRI sites.

#### pJJ

This vector has the hygromycin phosphotransferase selection gene *HptII* under control of the maize ubiquitin promoter. The maize ubiquitin promoter sequence with the ubiquitin 5′ intron was amplified from the pUbi-BASK vector using the primers UbiHEF and UbiOLR, and *HptII* was amplified from pGHyg [18] using the primers HygOLF and HygEcoRIR. The PCR products were digested with BamHI, and the resulting fragments ligated and used as a template for PCR with the UbiHEF and HygEcoRIR primers. This PCR product was digested with EcoRV and EcoRI and introduced into the SmaI and EcoRI sites of pCAMBIA0380. This construct lacks a nos terminator following the *HptII* sequence.

#### pJJ2LB

To build this construct, the Ubi-Bar-nos cassette was removed from pJJB2LB by digestion with EcoRI and HindIII and replaced with the Ubi-Hyg fragment from pJJ. This fragment is also lacking the nos terminator following the *HptII* gene.

#### pJJ2LBA

This construct contains a CaMV 4x35S activation tagging cassette and hygromycin selection under control of the maize ubiquitin promoter. The CaMV 4x35S enhancer fragment was derived from Ac-DsATag-BAR_gosGFP [49], in which it was flanked by two EcoRI restriction sites and contained one internal EcoRI. Ac-DsATag-BAR_gosGFP was digested with HindIII to release a fragment containing the CaMV 4x35S region and the Ubi-BAR-nos cassette, the ends of the fragment were blunted using mung bean nuclease and the fragment was then inserted into pCR4-TOPO. The ∼1.7 kb CaMV 4x35S enhancer fragment was released by partial digestion with EcoRI and introduced into the EcoRI site of pJJ2LB. Due to the repetitive nature of the CaMV 4x35S region, we were only able to sequence through three copies of the enhancer sequence after cloning into pJJ2LBA. However, enzymatic digestion confirmed the insert was of the expected size for four enhancer copies. This construct does not have a nos terminator following the *HptII* gene, but still transforms efficiently.

#### pJJ2LBP and pJJ2LBP2

These constructs were modified from pJJ2LB. Both contain hygromycin selection driven by the maize ubiquitin promoter and two gene trap reporters, a promoterless GUS gene (I2-GUS-T_n_) near the 2LB and a promoterless GFP gene (T_n_-sGFP-I3) near the RB. The gene trap cassettes were derived from the pGA2717 vector and contain rice tubulin intron sequences with splice donor and acceptor sites before the reporter gene sequences [41]. To make pJJ2LBP, the constitutive GUSPlus reporter and the RB were first removed from pJJ2LB by digestion with SphI. Primers (RB-f2 and RB-r2) were designed to amplify the pCAMBIA0305.2 right border and to introduce SpeI, AvrII and AseI restriction sites just before the RB and an SpeI site just following the RB sequence. This PCR fragment was introduced into the SpeI sites of pJJ2LB lacking the GUSPlus and original RB to create the intermediate pJJ2LB-RB2. Next, the I2-GUS-T_n_ cassette was released from pGA2717 by digestion with HpaI and KpnI, the ends were blunted, and the fragment cloned into the TOPO Blunt vector (Life Technologies, Grand Island, NY). The I2-GUS-T_n_ cassette was released from TOPO Blunt with EcoRI and ligated into pJJ2LB-RB2. Clones with the insert oriented with the I2 intron adjacent to the 2LB sequence were selected for the intermediate pJJ2LB-GUS-RB2. Next, the I3-sGFP-T_n_ cassette was amplified from pGA2717 using the GFP-f2 and GFP-r2 primers to introduce an AscI site before the I3 intron and an AvrII site after the nos terminator (T_n_). This fragment was inserted into the pJJ2LB-GUS-RB2 intermediate at the AscI and AvrII sites near the new RB to create the final pJJ2LBP vector. This construct is also lacking the nos terminator following the *HptII* gene. pJJ2LBP2 was made by the addition of a nos terminator to pJJ2LBP following the *HptII* gene. The nos terminator was amplified from pCAMBIA0305.2 with primers designed to add XmaI sites at the ends of the amplified DNA (nos-f and nos-r). The PCR fragment was introduced into the XmaI site between the *HptII* gene and the I2-GUS-T_n_ cassette of pJJ2LBP. The nos terminator was then checked for proper orientation.

### *Agrobacterium*-mediated Transformation

The *Agrobacterium*-mediated transformation protocol used to generate the T-DNA insertion lines in this collection was optimized from the protocol described in Vogel and Hill 2007 [18]. Embryos (<0.3–0.7 mm) were dissected from immature seeds and transferred to callus initiation media (CIM, per L: 4.43 g Linsmaier & Skoog basal medium (Phytotechnology, Shwanee Mission, KS #L689), 30 g sucrose, 1 ml 0.6 mg/ml CuSO_4_, pH 5.8. For plates, add 2 g phytagel (Sigma #P-8169). After autoclaving, add 0.5 ml of 5 mg/ml 2,4-D stock solution.) Following 3–4 weeks incubation in the dark at 28°C, embryogenic callus was subcultured onto fresh CIM plates. A second subculture was performed after two more weeks. The calluses from the second subculture were grown for one week before being used for transformation. On the day of transformation, calluses were bathed for 5 minutes in a suspension of *Agrobacterium* strain AGL1 containing the desired vector for transformation [54] (OD_600_ = 0.6) prepared in liquid CIM containing 200 µM 2,4-D and 0.1% Synperonic PE/F68 (Sigma #81112, formerly Pluronic F68). After removing as much of the *Agrobacterium* suspension as possible, the calluses were transferred to petri dishes containing a piece of sterile filter paper for co-cultivation for 3 days in the dark at 22°C. Note that co-cultivation under desiccating conditions is critical to the success of the transformation protocol. Next the callus pieces were moved to CIM plates containing 150 mg/L timentin and the appropriate selective agent to kill untransformed plant tissue - either 40 mg/L hygromycin B (Phytotechnology H397) or 60 mg/L DL-Phosphinothricin (Phytotechnology P679) - and incubated in the dark at 28°C for 1 week. Healthy sectors of hygromycin resistant transgenic callus were subcultured to fresh CIM plates one time for an additional two weeks of selection. BASTA selected callus was subjected to two additional rounds of subculture for an additional four weeks of selection. Note it is not necessary to obtain a callus with only healthy transgenic tissue because even small pieces of healthy callus surrounded by dead and dying callus will produce plantlets efficiently. Between 3 weeks (hygromycin selection) to 5 weeks (BASTA selection) after co-cultivation, calluses were transferred to regeneration media (per L: 4.43 g Linsmaier & Skoog (LS) basal medium, 30 g maltose, 2 g phytagel, pH 5.8; after autoclaving, 1.0 ml of sterile 0.2 mg/ml kinetin stock solution was added) containing 150 mg/L timentin and the appropriate selective agent. Plates were incubated in the light (cool-white fluorescent lighting at a level of 65 µEm^−2^ s^−1^ with a 16 hr light ∶ 8 hr dark cycle) at 28°C. Callus pieces began to turn green and shoots appeared between 2–4 weeks. Individual plantlets were moved to tissue culture boxes (we used sundae cups made for food service applications from Solo Corporation, Lake Forest, IL Cat. # SOL-TS5 (cups) and SOL-DL-100 (dome lids)) containing MS sucrose medium (per L: 4.42 g Murashige & Skoog (MS) basal medium with vitamins (Phytotechnology M519), 30 g sucrose, and 2 g phytagel, pH 5.7) and incubated in the light (cool-white fluorescent lighting at a level of 65 µEm^−2^ s^−1^ with a 16 hr light ∶ 8 hr dark cycle) at 28°C. After plantlets had formed roots and were approximately 2–5 cm tall, they were transplanted to soil and placed in a growth chamber for flowering (20 hr light, 4 hr dark, 24°C during the day and 18°C at night, cool-white fluorescent lighting at a level of 150 µEm^−2^ s^−1^). In this case, vernalization was not required to induce rapid flowering. Alternatively, to promote rapid flowering in plantlets moved directly to greenhouse conditions (no shading, 24°C in the day and 18°C at night with supplemental lighting to extend daylength to 16 h), plants were vernalized in tissue culture boxes or in soil under light (continuous cool-white fluorescent lighting, 4 µEm^−2^ s^−1^) at 4°C for 2–4 weeks depending on the season.

### PCR of empty donor sites in Ac/Ds lines

PCR was performed on DNA extracted from leaves of Ac-DsATag-Bar_gosGFP T-DNA lines. Primers HygBamHIF (5′ttggatccatgaaaaagcctgaactcacc3′) and HygKpnIR (5′ttggtaccctatttctttgccctcgg3′) were used to amplify the *HptII* gene. Locations of the primers R13pMOg22 (5′ggaaacgacaatctgatctctagg 3′) and Ac-promRev (5′ctcagtggttatggatgggagttg3′) that were used to identify the empty donor sites are shown as small black arrows in **Fig. 2A**.

### DNA Extraction

DNA extraction was based on the method described by Shiaman Chao and Daryl Somers (http://maswheat.ucdavis.edu). To prepare tissue, two young leaves (approximately 3 inches) from T_0_ plants were sampled into the wells of a 96-well plate (E&K Scienfitic, EK-22280). Glass or chrome steel beads (http://www.biospec.com) were added to the wells (either one 6.3 mm and two to three 3.5 mm glass beads or two 3.2 mm chrome steel beads per well), and the plates were lyophilized overnight. Directly after lyophilization, the plates were covered with sealing mats (E&K Scienfitic, EK-80080), and tissue was ground in a Retsch MM301 Ball Mill at 30 cycles/sec for 1 min. The grinding was repeated 5 times, for a total of 5 min. Before opening plates, the ground tissue was centrifuged at 4000 rpm for 20 min. at 4°C. To isolate DNA, 800 µl of extraction buffer (0.1 M Tris-HCl pH 7.5, 0.05 M EDTA pH 8.0, 1.25% SDS) preheated to 65°C was added to each well. Plates were sealed with a new sealing mat and shaken thoroughly. Chrome steel beads were removed prior to adding extraction buffer, but glass beads were left in the wells. To prevent contamination from neighboring wells, a flat weight was placed on top of the sealing mat and secured with several rubber bands. Plates were incubated at 65°C for 0.5–1 hour, with mixing every 5–10 min. Plates were transferred to ice for 15 min. before centrifuging at 4000 rpm for 5 min. at 4°C. Next, 400 µl cold 6M ammonium acetate was added to the wells, and the plates inverted several times and placed on ice for 15 min. Plates were then centrifuged at 4,000 rpm for 15 min. at 4°C to collect the precipitated proteins and plant tissue, and 900 µl of the supernatant was transferred into another plate containing 540 µl of isopropanol in each well. Plates were mixed thoroughly and placed on ice or at −20°C for 30 min. to precipitate DNA. Plates were then centrifuged at 4,000 rpm for 30 min. at 4°C, the supernatant decanted, and the plate inverted on a paper towel for a few seconds. Pellets were washed with 1 ml of 70% ethanol and centrifuged at 4,000 rpm for 20 min at 4°C. After removing the supernatant, the pellets were dried in the hood overnight and resuspended in 125 µl TE buffer (10 mM Tris-Cl pH 7.5, 1 mM EDTA pH 8.0). To dissolve the DNA, the samples were placed at 4°C overnight and then transferred to a 96-well microtiter plate. Estimated yield is 10–20 ng/µl.

### Inverse PCR

Restriction enzyme digestions were performed in 15 µl reactions containing 75–150 ng (7.5 µl) of DNA, 0.2 µl enzyme, and 1× NEB #1 buffer. Samples were incubated at 37°C for 3 hours, then at 65°C or 80°C (depending on enzymes) for 20 min. The enzymes used to digest individual T-DNA constructs at the right or left borders are listed in **Table 1**. Digestion products were purified by precipitation with 95% ethanol and 3M sodium acetate and resuspended in 10 µl of TE buffer (pH 8.0). Ligations were performed by adding 0.125 µl T4 DNA ligase, 1.0 µl of 10× ligation buffer, and 3.875 µl H_2_O to the digested DNA and incubating for 16 hr at 16°C. PCR reactions (10 µl) were prepared for the ligation products as follows: 2 µl 5× Go Taq buffer (NEB), 1 µl dNTPs (2.5 mM), 0.2 µl Primer1 (10 µM), 0.2 µl Primer2 (10 µM), 0.5 µl DMSO (100%), 0.05 µl Go Taq, 2 µl DNA (ligation), and 4.05 µl H_2_O. The PCR program used was 95°C for 5 min. and 94°C 30 sec, followed by 35 cycles (94°C 30 sec, 60°C 30 sec, 72°C 1 min. and 45 sec), and the program ended with 72°C 10 min. Some primer pairs needed 2–3 additional cycles to improve amplifications. If the PCR did not work well, another round of PCR was performed using nested primers and 0.5 µl of the first round PCR product as a template.

**Table 1:** Restriction enzymes and primers used for Inverse PCR.

Construct	Digestion	Border	PCR primers and annealing temperatures	Cycles*	Sequencing primer
pOL001	BfaI	LB	001-LB-F8R8, 62°C	38	001-LB-R6
			001-LB-F8R9, 57°C	38*	
			001-LB-F6R8, 60°C	35	
	BfaI	RB	001-SP3, 62°C	35	001-SP4
			001-RB-R6, 62°C	35	
	HhaI	LB	001-LB-F9R7, 60°C	35	001-LB-R9
	HhaI	RB	001-SP3, 62°C	35	001-SP4
			001-RB-R6, 62°C	35	
	TaqI	RB	001-SP3, 62°C	35	001-SP4
			001-RB-R6, 62°C	35	
pJJ2LB	HpyCH4IV	LB	pJJ2LB -LB-F4R2, 60°C	35	UH2LB-LB-R5
	HpyCH4IV	RB	pJJ2LB -RB-F1R1, 53°C	35	UH2LB-RB-F3
	BfaI	LB	pJJ2LB -LB-F1R2, 60°C	35	UH2LB-LB-R5
	TaqI	RB	pJJ2LB -RB-F1R1, 53°C	35	UH2LB-RB-F3
pJJ2LBP2	BfaI	LB	pJJ2LBP2-LB-F3R3, 60°C	35*	PJJ2LBP2-LB-R1
			pJJ2LBP2-LB-F3R1,60°C	35	PJJ2LBP2-LB-R2
	BfaI	RB	pJJ2LBP2-RB-F1R1, 63°C	35	PJJ2LBP2-RB-F3
	HpyCH4IV	LB	pJJ2LBP2-LB-F5R5, 50°C	35	PJJ2LBP2-LB-R3
	HpyCH4IV	RB	pJJ2LBP2-RB-F2R3, 50°C	37	PJJ2LBP2-RB-F3
	TaqI	RB	pJJ2LBP2-RB-F2R3, 50°C	37	PJJ2LBP2-RB-F3
pJJ2LBA	HpyCH4IV	LB	pJJ2LBA-LB-F2R1, 58°C	35	PJJ2LBA-LB-R2
			pJJ2LBA-LB-F1R1, 58°C	35	
	HpyCH4IV	RB	pJJ2LBA-RB-F1R1, 60°C	35	PJJ2LBA-RB-F3
	HpaII	RB	pJJ2LBA-RB-F1R1, 60°C	35	PJJ2LBA-RB-F3

* indicates PCR primers requiring a second round of PCR.

### FST Sequencing

Unconsumed dNTPs and primers were removed from PCR products using ExoSAP-IT (Affymetrix) per manufacturer's instructions. Sequencing was performed in either 96 (10 µl reactions) or 384 (5 µl reactions) well plates with the BigDye Terminator v3.1 sequencing kit (Applied Biosystems) using the following program: 98°C 5 min, followed by 39 cycles (96°C 10 sec, 50°C 10 sec, 60°C 4 min), and holding at 4°C. The sequencing products were precipitated with 95% ethanol and 3M sodium acetate, washed with 70% ethanol, dried in a hood (hours to overnight). The pellets were dissolved in 8.5 µl (for a 96 well plate) or 5 ul (for a 384-well plate) of Hi-Di Formamide (Applied Biosystems), and the plates stored at −20°C until use. Before loading the sequencer, the samples were denatured at 96°C for 3 min. Primer locations and sequences are listed in **Table 2**.

**Table 2:** Sequences and locations of primers used for Inverse PCR.

Name	Sequence (5′-3′)	Location
pOL001-LB-F6	GACCCGGTCGTGCCCCTCT	8548-8566
pOL001-LB-R6	TTAAAAACGTCCGCAATGTGTTATTAAG	8484-8457
pOL001-LB-F8	TGCCTGCAGTGCAGCGTGACC	8531-8551
pOL001-LB-R8	TTCAGTACATTAAAAACGTCCGCAATGTG	8493-8465
pOL001-LB-R9	GATAAGCTGTCAAACATGAGAATTCAG	8515-8489
pOL001-SP3	CGTCATCGGCGGGGGTCATAAC	14003-14024
pOL001-SP4	TCTCCGCTCATGATCAGATTGTCG	14039-14062
pOL001-RB-R6	AAGCACATACGTCAGAAACCATTATTGCG	13915-13887
pJJ2LB -LB-F1	CTCGCTAAACTCCCCAATGTCAAG	901-924
pJJ2LB -LB-F4	ACAGCGGGCAGTTCGGTTTCA	824-844
pJJ2LB -LB-R2	CTCGTCCGAGGGCAAAGAAATAGG	152-129
pJJ2LB -LB-R5	TCCTGTGTGAAATTGTTATCCGC	103-81
pJJ2LB -RB-F1	ACGCGATAGAAAACAAAATATAGC	5664-5687
pJJ2LB -RB-R1	GCGGGACTCTAATCATAAAAACC	5626-5648
pJJ2LB -RB-F3	GCGCAAACTAGGATAAATTATCG	5688-5710
pJJ2LBP2-LB-F3	CTCTACACCACGCCGAACACCTG	793-815
pJJ2LBP2-LB-R3	CCTGTGTGAAATTGTTATCCGCTCAC	102-77
pJJ2LBP2-LB-R1	CACAACATACGAGCCGGAAGCATA	69-46
pJJ2LBP2-LB-F5	GTGGCTAATTACATGACTAACTTGG	182-206
pJJ2LBP2-LB-R5	ATAGCACCGTGGTAGTAAGAATG	147-169
pJJ2LBP2-RB-F1	GCTCCTCGCCCTTGCTCACCAT	6221-6242
pJJ2LBP2-RB-R1	TGTTGCCGGTCTTGCGATGATTATC	5460-5436
pJJ2LBP2-RB-F3	GCAGTGAATTAACATAGCAGAGAA	6355-6378
pJJ2LBP2-RB-F2	ACAAACAAGAAATGGCAGTGAAT	6341-6363
pJJ2LBP2-RB-R3	TCTAACCAACTTGTTTATTGCTAATG	6333-6308
pJJ2LBA-LB-F1	ACAGGAAACAGCTATGACATGATTACGA	98-125
pJJ2LBA-LB-R1	ACACAACATACGAGCCGGAAGCATA	46-70
pJJ2LBA-LB-R2	GAGCCGGAAGCATAAAGTGTAAAG	36-59
pJJ2LBA-RB-F1	TCGAGAGGGGTCCAGAGGCA	4053-4072
pJJ2LBA-RB-R1	CTGTCGGCATCCAGAAATTGCG	4001-4022
pJJ2LBA-RB-F3	AGATGCCGTGCCGTCTGCT	4080-4098

### GUS staining of JJ2LBP and JJ2LBP2 lines

Stem, leaf and floral tissue samples were collected from young *Brachypodium* plants into microcentrifuge tubes. GUS staining solution (0.1 M sodium phosphate pH 7.0, 0.5 mM potassium ferrocyanide, 0.5 mM potassium ferricyanide, 0.5% v/v Triton X-100, 0.15% w/v X-Gluc) was added directly to the tubes, and samples were vacuum infiltrated for 5 min before placing at 37°C in the dark overnight. The GUS staining solution was removed with a pipet and 95% EtOH was added to remove any chlorophyll that might mask the blue staining and to fix the tissue.

## Results

### Optimization of transformation

We performed a series of experiments to improve transformation efficiency, defined as the number of transgenic plantlets regenerated per number of callus pieces co-cultivated with *Agrobacterium*, and decrease the labor required per transgenic plant produced. The following modifications were found to improve transformation efficiency. The production of high quality embryogenic callus was increased by the addition of copper sulfate at a final concentration of 0.6 mg/L to the callus initiation media [19]. Callus pieces were moved to media containing the selective agent (hygromycin or Basta) directly after the 3 day co-cultivation step, rather than after a 7 day recovery on media lacking the selective agent. This modification permitted us to eliminate one of the subculture steps after co-cultivation decreasing labor and supplies and accelerating the recovery of transgenic plants. After rooting, the regenerated plantlets were placed at 4°C for two to three weeks before moving them into soil to cue early flowering in the greenhouse and bypass the need for growing the plants under long days in a growth chamber. *Brachypodium* accessions Bd21-3 and Bd21 were compared as the source of embryogenic callus. Transformation efficiency was similar for the two lines when a microscope was used to identify embryogenic callus during subculture. However, Bd21-3 was selected for the production of our insertional mutant population, because it forms a more strongly yellow colored callus with organized structures that eased selection of the correct callus and increased transformation efficiency when subculture was performed without the aid of a microscope.

Optimization of the transformation vector was responsible for the greatest improvement in transformation efficiency. We conducted experiments to compare the recovery of transgenic plants using hygromycin or Basta selection under the control of three different promoters (**Table 3**). The pOL001 vector previously was used to produce transgenic *Brachypodium* with transformation efficiency up to 41% [18]. This construct contains hygromycin selection under the control of the CaMV 35S promoter and was used as a benchmark in all transformation optimization experiments. Three additional hygromycin selection vectors and three vectors designed with Basta selection were compared with pOL001. The pGA2717 rice transformation vector [41] contains hygromycin selection driven by the rice tubulin promoter. Two additional vectors, pJJ and pJJ2LB, were constructed by placing hygromycin selection under control of the maize ubiquitin promoter and inserted into pCAMBIA0305.2 (http://www.cambia.org/). The pJJ vector utilizes the single left border (LB) from pCAMBIA0305.2, but in the pJJ2LB vector, the pCAMBIA0305.2 LB was replaced with a double left border from the pL3 vector [53]. The pJJB and pJJB2LB vectors were constructed similarly, but have the maize ubiquitin promoter driving Basta selection. The final vector, pSMAb801 has the CaMV 35S promoter directing expression of the Basta selection gene [55].

**Table 3:** T-DNA constructs evaluated for transformation efficiency.

Construct	Average Efficiency*	Promoter	Selection	LB copies
pOL001	22.9	CaMV 35S	hygromycin	1
pJJ	55.8	Maize ubiquitin	hygromycin	1
pJJ2LB	36.5	Maize ubiquitin	hygromycin	2
pGA2717	0.7	Rice tubulin	hygromycin	1
pJJB	3.0	Maize ubiquitin	Basta	1
pJJB2LB	8.3	Maize ubiquitin	Basta	2
pSMAb801	2.2	CaMV35S	Basta	1

* Efficiency is calculated as the percentage of callus pieces co-cultivated with *Agrobacterium* that go on to produce transgenic plants.

Transformation efficiency was evaluated for these seven vectors in multiple experiments (**Table 3**). Our results show that transgenic plants can be recovered using either hygromycin or Basta as the selective agent, however, vectors containing hygromycin selection, with the exception of vector pGA2717, were much more efficient (averages 22.9 to 55.8%) than those employing Basta selection (averages 2.2 to 8.3%). The hygromycin-selected pGA2717 vector yielded the lowest transformation efficiency observed, 0.7%, possibly due to the function of the rice tubulin promoter. However, since we did not sequence the vector we cannot rule out the possibility that there was something wrong with the construct. Of the plantlets regenerated on hygromycin, 83.5% survived to produce T_1_ seed whereas only 47.1% of the plantlets regenerated on Basta survived (data not shown). Furthermore, the strong selection of hygromycin permitted transfer of callus to regeneration media after three weeks of selection rather than five, accelerating the time to recovery of transgenic plants compared to Basta selection. The addition of a second left border did not affect plant generation or survival (data not shown).

Additional experiments compared transformation efficiency for pOL001 and pJJ2LB. Hygromycin selection is driven by different promoters in these two vectors (CaMV 35S in pOL001 and maize ubiquitin in pJJ2LB). In six side by side transformation experiments 1,037 pieces of callus were co-cultivated with *Agrobacterium* carrying pOL001 and 991 pieces were co-cultivated with *Agrobacterium* carrying pJJ2LB. Transformation efficiency was significantly higher for pJJ2LB containing the maize ubiquitin promoter (48%) than for pOL001 containing the CaMV 35S promoter (32.2%). For both of these constructs, more than 95% of the plants tested were positive for GUS reporter gene expression and greater than 90% of the regenerated plantlets survived to set T_1_ seed (data not shown). Using our optimized method and vectors, transformation efficiencies averaged 42% during the production of the mutant population and efficiencies of 50–75% were achieved for individual experiments.

### Generation of T-DNA lines

The bulk (82.6%) of the T-DNA population was generated from three constructs pJJ2LB, pJJ2LBA and pJJ2LBP2 (**Fig. 1**). All three constructs were designed with two left borders to try to limit the transfer of vector DNA beyond the left border [51]. Constructs pJJ2LB and pJJ2LBP2 can only affect gene function by insertion into coding or regulatory regions. In addition, pJJ2LBP2 can function as a gene trap to identify adjacent promoters because it contains reporterless GUS and GFP genes with multiple splice acceptors at the left and right borders respectively [41]. In addition to disrupting gene function by insertion, the pJJ2LBA can act as an activation tag that causes the overexpression of nearby genes because it contains four copies of the CaMV 35S enhancer sequence adjacent to the LB [43], [56]. Overall, 8,491 fertile lines were produced that comprise the WRRC *Brachypodium* T-DNA insertional mutant population described in this report (**Fig. 1B** **and Table S2**). Since this is an ongoing project, additional lines continue to be produced and readers are directed to the project website (http://brachypodium.pw.usda.gov/) for the most up to date totals.

### Evaluation of transposon tagging in *Brachypodium*

To determine if transposon tagging could be used to generate insertional mutants in *Brachypodium* efficiently, we tested two transposon systems (**Fig. 2**) that have been used previously for large-scale mutagenesis in rice and Arabidopsis, Ac/Ds and EnSpm [57], [58]. *Agrobacterium*-mediated transformation was used to deliver T-DNAs containing the transposon constructs. Both T-DNAs contain hygromycin selection for plant transformation, a GFP reporter gene, and a transposase. In addition, each construct contains a mobile element harboring four copies of the CaMV 35S enhancer sequence for activation tagging. The average transformation efficiency of Ac-DsATag-Bar_gosGFP (Fig. 1A) [49] over six transformations was 27.2%, but most plants either died before setting seed or produced non-viable seed (**Fig. 2B**). Only 5.9% (7 of 119) of the Ac/DsAtag-Bar_gosGFP T_0_ transgenic plants survived to set T_1_ seed. PCR was performed on genomic DNA extracted from eight Ac/DsAtag-Bar_gosGFP T_0_ plants (**Fig. 2C**). All samples tested positive for the presence of the *HptII* gene indicating the T-DNA was present in the plants. Transposition of the Ds element leaves behind an empty donor site that can be detected when PCR is performed with primers located outside of the Ds region (**Fig. 2A**). If the Ds element remains in place, the distance between the primers is too large to amplify a fragment. Five of the 8 plants tested for an empty donor site yielded a band with a size indicating that the Ds element had moved (**Fig. 2C**) and none of these lines set seed. In the case of line 171-3, the larger PCR fragment suggests that the Ds excision was incomplete. Line 175, one of the lines in which the Ds element did not move, is also a line that produced T_1_ seed. We conclude the Ac/Ds transposon functions in *Brachypodium*, but is lethal, possibly because it is too active. Similarly, transformations with a construct containing dSpm (pdSpm-R) [50] yielded 22 plantlets, all but one of which died while still very small (**Fig. 2D**). However, all callus pieces transformed with pdSpm-R displayed GFP fluorescence indicating that they were transformed with the construct (**Fig. 2E**). Although there is potential for optimization of transposon systems for the production of mutants in *Brachypodium*, we chose to focus on T-DNA tagging to generate insertional mutants.

### Expression of ß–glucuronidase (GUS) from the pJJ2LBP and pJJ2LBP2 gene trap vectors

**Figure 3:**
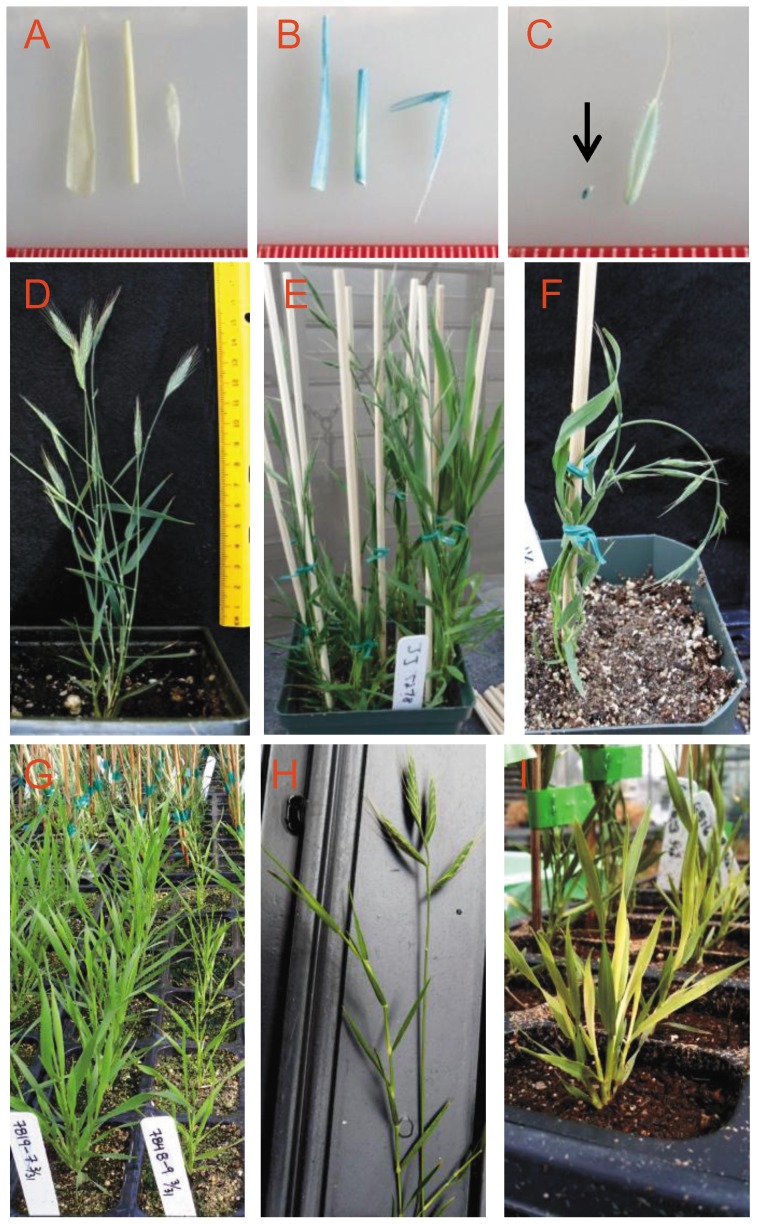
Phenotypes observed in the T-DNA population. **A–C.** GUS staining of lines created with pJJ2LBP and pJJ2LBP2 vectors. Leaf, stem and floral tissue (arranged left to right) collected from plants transformed with gene trap vectors were stained to detect GUS expression. Ruler divisions are in millimeters. **A.** Blue color was not detected in line JJ5896 indicating an absence of GUS expression. **B.** GUS expression is indicated by the blue color in all three tissue types for line JJ6405. **C.** For line JJ6135 expression was primarily detected in the developing embryo indicated by the black arrow. **D–I.** Images highlighting a selection of mutant phenotypes observed in the T-DNA population. **D.** Wild-type Bd21-3 plant. **E.** Flowering time variation illustrated by a late flowering mutant in the front right of the pot. **F.** Morphology variation represented by a mutant with curling stems and leaves. **G.** Size differences illustrated by the row of short plants on the right. **H.** Increased segmentation, branching and delayed flowering time (left) compared to the wild-type plant (right). **I.** Pigmentation mutants.

To assess the function of the gene trap vectors, 235 lines containing pJJ2LBP and 500 lines containing pJJ2LBP2 lines were assayed for GUS activity as an indicator that the promoterless GUS reporter gene was being expressed by a *Brachypodium* promoter. Since the T_1_ generation examined was segregating for the transgenes, we first used PCR to identify transgenic T_1_ plants by amplifying the *HptII* gene contained on the T-DNA (data not shown). Leaf, stem, and floral samples for each transgenic plant were placed in a GUS staining solution and incubated overnight. Upon visual examination of cleared tissue, 53 (7.2%) of the lines showed GUS expression in at least one tissue type (**Fig. 3A, B, C**). Seven lines showed blue staining in all three tissues sampled (**Fig. 3B**), 14 lines in vegetative tissues only (leaf and stem), and 22 lines in floral tissue only (**Fig. 3C**). The remaining 10 positive lines had GUS expression in flowers and either stem or leaf tissue. Seeds for 95 lines were germinated on MS media containing hygromycin and tested for GUS expression in roots, but blue staining was not detected in the roots (data not shown). Using the vector from which we derived our gene trap cassettes, pGA2717, Ryu et. al. observed GUS staining in 4.8% of 3,140 rice lines [41], a value similar to what we observed in *Brachypodium*. Another study of 8,200 rice lines using a similar vector, pTAG8, reported GUS staining in vegetative tissue for 11% of the lines tested and in reproductive tissue for 22% of the lines tested [42]. These results demonstrate that the GUS reporter derived from the pGA2717 rice gene trap vector functions in *Brachypodium* and suggest there is room for optimization of gene trap vectors for *Brachypodium*.

### Phenotypes observed in T-DNA insertional mutant lines

T_1_ seeds were planted for nearly 2,000 mutant lines for which insertions had been identified within or near genes (see below for details on insertion sites). The T_1_ plants represent the first generation segregating for T-DNA insertions and provided the opportunity to survey for mutant phenotypes and to bulk seeds. Phenotypes were noted in approximately 5% of the lines planted. Classes of phenotypes included: size mutants such as tall, short, or dwarf plants (35 lines); early and late flowering time mutants (28 lines); pigmentation mutants such as albinos, variegated leaves, and mottled leaves (25 lines); weak or sickly plants (5 lines); mutants with altered morphology, including curling stems and leaves or branching variations (8 lines); and fertility mutants (3 lines). A few examples of these phenotypes are shown in **Figure 3**.

### Generating flanking sequence tags using inverse PCR

**Figure 4:**
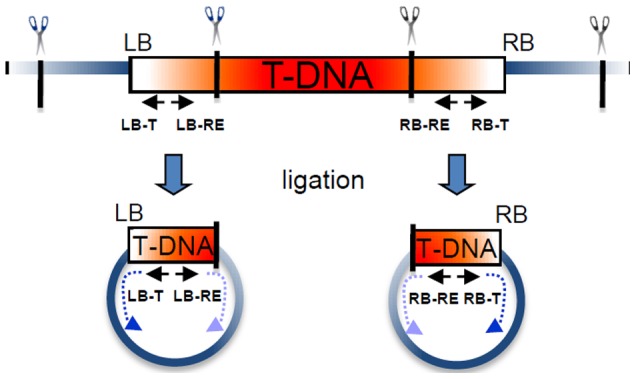
IPCR strategy for obtaining T-DNA flanking sequences. The diagram illustrates the IPCR strategy used to obtain sequences flanking the T-DNA insertion sites (ISs). Restriction enzymes (shown as scissors) with recognition sites (black lines) near the T-DNA border sequences (LB and RB) were used to digest DNA from T-DNA insertion lines. Enzymes cut both within the T-DNA and genomic sequence, and ligations were performed to circularize purified digestion products. PCR of the ligation products was performed using T-DNA specific primers and sequencing was performed with nested primers. The orientations and locations of primers with respect to restriction sites are shown as black arrows. Primers located within the T-DNA directed toward the junction with genomic DNA are designated T primers (LB-T and RB-T). Sequencing reactions using these primers return genomic sequence directly adjacent to the IS (dark blue dotted arrows). Primers directed into the T-DNA are designated RE primers (LB-RE and RB-RE). After enzyme digestion and ligation, sequencing reactions using these primers return genomic sequence starting from the closest restriction enzyme recognition site within the *Brachypodium* genomic sequence and directed toward the T-DNA insertion (light blue dotted arrows).

Inverse PCR (IPCR) of genomic DNA from individual transgenic plants was used to obtain sequences adjacent to the T-DNA insertions in mutant lines. Sequences that match the *Brachypodium* genome serve as flanking sequence tags (FSTs) and define the locations of the T-DNA insertion sites (ISs). The IPCR method (**Fig. 4**) depends on restriction enzyme digestion and the ligation of a digestion site within the T-DNA to the nearest genomic digestion site for the same enzyme [57], [58]. To determine the best means of recovering FSTs, we performed sequencing reactions from both the LB and the RB of the T-DNA. Multiple enzymes and sequencing primers were also tested. Primers oriented out of the T-DNA (designated T primers) yield sequences directly adjacent to the T-DNA and are directed into the flanking genomic region. Primers oriented into the T-DNA (designated RE primers) result in sequences starting at the genomic restriction enzyme site and are directed toward the T-DNA (**Fig. 4**).

Initial studies tested the efficiency of recovering FSTs after digestion with the enzyme HpyCH4IV. This enzyme was chosen because it is located near both of the T-DNA borders, and therefore, one digestion and ligation reaction could be used for four sequencing reactions. Sequencing from the LB using T primers (LB-T) returned FSTs for 232 of 567 lines tested (40.9%), and reactions from the LB using RE primers (LB-RE) produced FSTs for only 189 of the same 567 lines (33.3%). Similar results were observed when sequencing from the RB. Reactions using T primers (RB-T) returned FSTs for 178 of 378 lines tested (47.1%) and reactions using the RE primers (RB-RE) reactions produced FSTs for 172 of 474 lines tested (36.3%). These results indicate that at both borders, sequencing directly from the T-DNA into the genomic sequence using T primers was more efficient at generating FSTs than sequencing from the genomic restriction site back toward the T-DNA using RE primers. In all four sets of reactions described above (LB-T, LB-RE, RB-T, and RB-RE), the majority of the sequences recovered contained vector sequences (51–61%), and 7–15% of the sequences failed to match any known sequence or were not readable due to low quality scores. A comparison of early sequencing reactions from the pJJ vector derivatives showed that the addition of a second LB did not improve the efficiency of FST recovery (data not shown).

In an effort to increase the efficiency of recovering FSTs, IPCR was conducted using two enzymes adjacent to the RB of the pJJ vectors. The two enzymes, HpyCH4IV and HpaII, were used in RB-T reactions for 852 T-DNA lines. HpyCH4IV digestion resulted in FSTs for 350 lines (41.1%) and HpaII digestion returned FSTs for 344 lines (40.4%). In combination, FSTs for 433 lines (50.8%) were obtained from the IPCR reactions after digestion with the two enzymes. The two enzyme approach increased the efficiency of obtaining FSTs from the RB approximately 25% over the single enzyme approach. When LB-T reactions were included for the HpyCH4IV digestion, the number of lines with FSTs reached 531 (62.3%), further increasing the efficiency by more than 20%.

In a separate set of tests, FST recovery was evaluated when IPCR reactions were performed after digestion with three different enzymes. Using BfaI in LB reactions, we obtained FSTs for 38.6% of the lines tested. In HpyCH4IV reactions from the LB and RB, we recovered FSTs for 55.3% of the lines. Together, these two enzymes yielded FSTs for 66.4% of the lines tested. Addition of IPCR reactions from the RB using a third enzyme, TaqI, only increased the total to 67.4% of the lines tested. As a result of these experiments, we decided to use the two borders, two enzymes approach to obtain FSTs for our T-DNA lines.

### Identifying flanking sequence tags and assigning insertion sites

Data from 17,637 inverse PCR sequencing reactions for 7,145 T-DNA lines were compared to the *Brachypodium distachyon* genome assembly v1.0 using BLASTn (**Table 4**). To maximize the detection of T-DNA insertion loci, we assigned an e-value cutoff of 10^−3^. Using this criterion, 7,389 sequences (41.9%) matched the *Brachypodium* genome. These sequences represent 4,402 (61.6%) of the lines analyzed. The remaining 10,248 sequences that failed to match the *Brachypodium* genome were vector sequences, sequences with no identified matches, or poor quality sequences. The top scoring BLAST hit for each FST was used for subsequent analysis. The only exceptions were FSTs that exactly matched more than one location in the genome (discussed below). The average FST length was 195 bases and the median length 142 bases. We defined the base of the FST closest to the T-DNA to be the insertion site (IS). However, since we often did not sequence across the junction, the actual IS may differ slightly. Multiple sequencing reactions were performed for many of the T-DNA lines using T and RE primers that anneal near the RB or LB. Since independent reactions may return different sequences for a single line, a particular line can have FSTs assigned to more than one location in the genome. This is not surprising because the average number of T-DNA insertions per line is ∼2 [18]. When a single line has multiple FSTs, they are distinguished by the addition of a numerical suffix to the name of the T-DNA line. For example, four sequencing reactions were performed for the T-DNA line JJ3, yielding four FSTs. The FSTs were designated as JJ3.0, JJ3.1, JJ3.2, and JJ3.3. The JJ3.0 FST is located on chromosome 1, and the other three FSTs are assigned to chromosome 2. When a T-DNA line has more than one FST located on the same chromosome within a 1 kb range, the FSTs were treated as a single IS. For example, JJ3.1 and JJ3.2 are both located on Bd2 separated by only 284 bases, and therefore are counted as one T-DNA IS. Using this classification, the 7,389 FSTs in this collection represent 5,285 distinct ISs. Of these sites, 1,538 (29.1%) ISs are supported by more than one FST (in 1,501 lines) (**Tables 5** **and** **6**).

**Table 4:** Flanking sequence tags (FSTs) generated for T-DNA lines.

Sequences	Number of sequences	Percentage of total sequences
Generated	17,637	100.0
BLASTed vs *B. distachyon* genome	17,599	99.9
Matches to *B. distachyon* genome	7,389	42.0
Unique insertion sites (IS)	5,285	30.0

Lines	Number of lines	Percentage of lines
Sequenced	7,145	100.0
FST assigned	4,402	61.6
No FST assigned	2,743	38.4
FST assigned to sterile lines	251	5.7

**Table 5:** Number of T-DNA integration events detected per line.

IS detected/line	Number of lines	Percentage of lines with FSTs
1	3,629	82.4
2	678	15.4
3	84	1.9
4	8	0.2
5	2	<0.1
6	1	<0.1
**Total**	4,402	100

**Table 6:** Number of FSTs supporting individual ISs.

FST/IS	Number if ISs	Percentage of ISs
1	3,747	70.9
2	1,109	21.0
3	315	6.0
4	95	1.8
5	15	0.3
6	4	<0.1
**Total**	5,285	100

In the majority of the T-DNA lines with assigned FSTs, one (82.4%) or two (15.4%) ISs were identified (**Table 5**), and fewer than 3.0% of the lines were assigned three or more ISs. In cases where the T-DNA has inserted into a repetitive sequence, the BLAST search returned multiple hits with equal scores and prevented assignment of an unambiguous IS. We observed this for 0.6% of the lines (28 lines) with FSTs. There are two primary locations in the genome where these insertions mapped. One line (JJ4195) returned a sequence that was assigned to 29 loci near the centromere of chromosome Bd4 in a region that contains *Brachypodium* centromeric retroelements, and 27 lines gave sequences that were assigned to 649 loci in the first 215 kb of the short arm of Bd5 in a region encoding 26S ribosomal RNA genes. To simplify our analyses we only used one IS for each FST. For the majority of the T-DNA lines, the single FST assigned from the top scoring BLAST hit for each sequencing reaction is displayed on the USDA-ARS-WRRC T-DNA website (described in a later section). However, for the insertions in repetitive regions, each of the potential genomic locations for the equal scoring BLAST hits is displayed.

### Distribution of ISs within the *Brachypodium* genome

**Figure 5:**
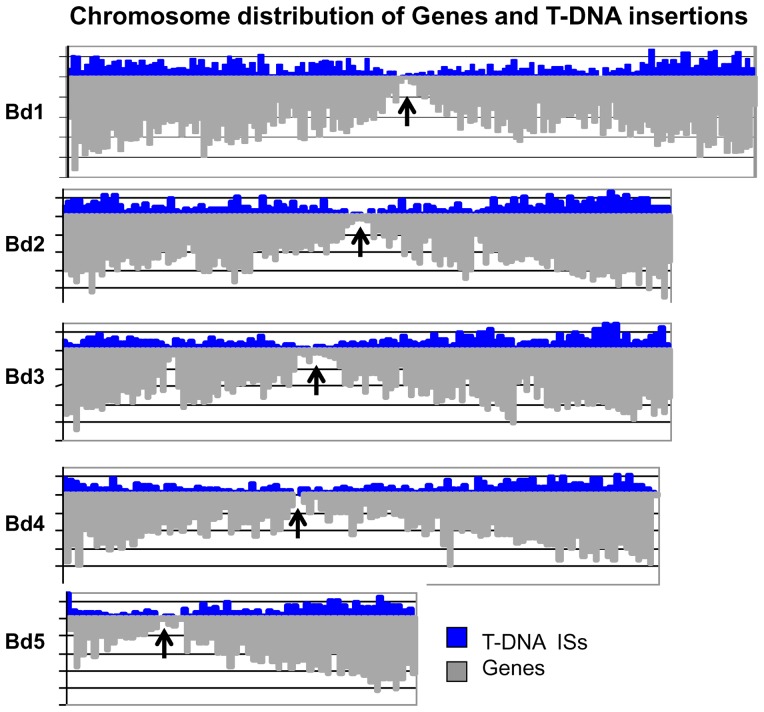
Distribution of Genes and T-DNA insertions along the chromosomes. T-DNA insertions (blue) and Brachypodium genes (gray) are plotted in 500 kb windows moving across the length of each chromosome. Each division on the Y-axis represents 20 insertions sites or genes, respectively. Chromosome numbers are listed to the left. Insertions span the length of all five chromosomes and are positively correlated with genic regions. A greater density of insertions is observed near the distal ends of the chromosomes than is observed near the centromeres (approximate locations indicated by black arrows).

The distribution of ISs across the five *Brachypodium* chromosomes was analyzed by plotting the number of insertions within 500 kb windows moving across the length of each chromosome starting from the beginning of the short arm to the end of the long arm (**Fig. 5**). Insertions span the entire length of all five chromosomes. For chromosomes 1, 2, 3, and 5, the number of insertion sites detected per chromosome is generally proportional to the chromosome length and the distribution ranges from 19.2 to 20.9 ISs/Mb with an average of 20.2 ISs/Mb (**Table 7**). Fewer ISs were detected on chromosome 4 relative to its size (15.9 ISs/Mb) than were observed for the other chromosomes and results in an average over the entire genome of 19.3 ISs/Mb. Overall, the number of genes/Mb is directly proportional to the length of the chromosome. Thus, the lower number of ISs/Mb on chromosome 4 may be partly attributable to the lower number of genes/Mb on this chromosome (**Table 7**). A non-uniform distribution of ISs similar to that reported for T-DNA insertions in rice [57]–[59], *Arabidopsis* [60], and the BrachyTAG [48] collection also is observed in the WRRC population. In general, the distal ends of the chromosomes have a greater density of insertions, and fewer insertions are detected near the centromeric regions (**Fig. 5**). The *Brachypodium* v1.0 annotation [8] was used to plot the number of genes present in the same 500 kb windows used to visualize the distribution of ISs. Higher numbers of ISs correlate well with the regions of higher gene density (**Fig. 5**).

**Table 7:** Chromosome distribution of FSTs, ISs, and genes.

Chromosome	Mb	# FSTs		FST/Mb	ISs	ISs/Mb	genes/Mb
Bd1	74.8	2,201		29.4	1,568	20.9	99.8
Bd2	59.3	1,738		29.3	1,234	20.8	97.6
Bd3	59.9	1,601		26.7	1,148	19.2	94.1
Bd4	48.6	1,092		22.5	774	15.9	86.1
Bd5	28.3	757		26.7	561	19.8	85.9
**Total**	**271**	**7,389**	**Avg.**	**26.9**	**5,285**	**19.3**	**92.7**

### Distributions of FSTs in genic and intergenic regions

**Figure 6:**
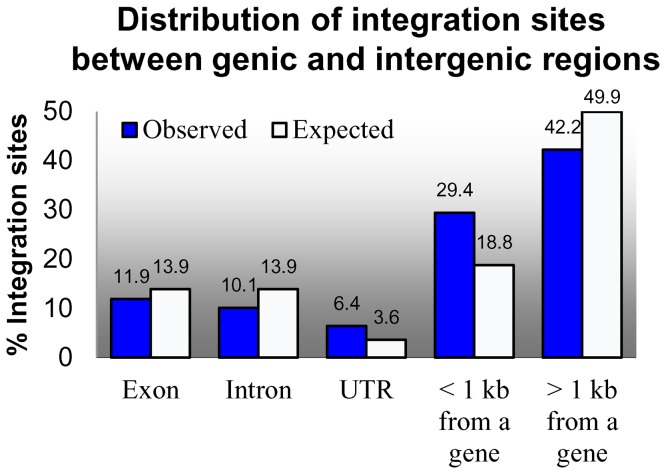
Distribution of insertion sites between genic and intergenic regions. A comparison of the observed and expected percentages of T-DNA insertions is illustrated for each insertion class. Insertions in UTRs and within 1 kb of a gene are observed to be substantially higher than expected. Intergenic insertions >1 kb from a gene are lower than expected.

To analyze the T-DNA distribution between genic and intergenic regions (**Fig. 6** **and** **Table 8**), we compared the assigned IS loci to the v1.0 annotation reported by the International Brachypodium Initiative [8]. The report identified 25,532 protein coding genes in the *Brachypodium* genome with an average gene length of 3,336 bases, including exons, introns, and UTRs. This represents 31.3% of the 272 Mb genome. In our population, 28.4% of the ISs (1,499) reside within genes, a value slightly lower than the percentage of the genome assigned to genes. To more precisely describe the ISs we further categorized insertions into genes as insertions into exons, introns, and UTRs. We estimate the percentage of the genome represented by each of these classes as 13.9% exon sequences, 13.9% introns and 3.6% UTRs. It should be noted that UTRs are significantly underestimated because sequences were only annotated as UTRs if there was EST support for the UTR. Using these estimates, we observed a lower number of T-DNA insertions in exons (11.9%) and in introns (10.1%) compared to what would be expected from the percentages of the genome represented by these features. This is in accordance with the lower than expected proportion of T-DNA insertions observed for whole genes. In contrast, the 6.4% (338) of the T-DNA insertions assigned to UTRs is nearly twice the amount expected (3.6%) based on the percentage of the genome assigned to UTRs. In our population, T-DNA insertions slightly favored 3′ UTRs (191, 3.7%) over 5′ UTRs (147, 2.6%). We were also interested in identifying insertions near genes, because sequences adjacent to genes often encode elements critical for the regulation of gene expression. To do this, we defined an IS to be near a gene if it was located within 1 kb of the 5′ or 3′ end of a gene model included in v1.0 annotation of the *Brachypodium* genome. The 2,000 bases flanking each of the 25,532 *Brachypodium* genes represent 18.8% of the genome. Of the ISs in intergenic space, 29.4% (1,556) are present within 1 kb of gene sequences. Similar to what is observed for UTRs, the integration of T-DNAs near genes is significantly higher than the proportion expected, but in this case, T-DNA insertions were more often observed 5′ of genes (931, 59.2%) than 3′ of genes (625, 40.8%).

**Table 8:** Distribution of FSTs and ISs between genic and intergenic regions.

Insertion class	Number of FSTs	Number of ISs	Percentage of ISs	Expected percentage	Difference from expected
Insertions in genes (intron, exon, UTR)	2,025	1,499	28.4	31.3	−2.9
Exon	855	629	11.9	13.9	−2.0
Intron	705	532	10.1	13.9	−3.8
UTR	465	338	6.4	3.6	2.8
5′ UTR	192	147	2.8	-	-
3′ UTR	273	191	3.6	-	-
Intergenic insertions <1 kb from a gene	2,203	1,556	29.4	18.8	10.6
5′ of gene	1327	931	17.6	-	-
3′ of gene	876	625	11.8	-	-
Intergenic >1 kb from a gene	3,161	2,230	42.2	49.9	−7.7
Total	7,389	5,285			

### Online resources for accessing the USDA-ARS Western Regional Research Center (WRRC) T-DNA collection

The goal of this project was to add to the growing collection of genomic resources available for *Brachypodium* by creating a large collection of sequence-indexed T-DNA lines. Mutant lines are available to interested researchers through a link from the *Brachypodium* resource page of the Genomics and Gene Discovery Research Unit at the USDA-ARS, WRRC (http://brachypodium.pw.usda.gov/). The WRRC T-DNA website (http://brachypodium.pw.usda.gov/TDNA/) includes a GBrowse window for visualization of FSTs in the context of adjacent genomic features and a window for BLAST searches against the regions adjacent to T-DNA insertion sites. In addition, an Excel table listing FST details (**Table S2**) and FASTA file containing the genomic regions flanking T-DNA ISs (**File S1**) are available for download. Instructions for ordering lines are provided. In addition, the WRRC T-DNA collection can be viewed as a track in the http://www.brachypodium.org/ Gbrowse window.

## Discussion

By modifying our transformation protocol we were able to significantly increase transformation efficiency and reduce the length of time necessary to generate transgenic plants. Specifically, we eliminated the recovery step where callus was placed on callus inducing media without hygromycin for a week after co-cultivation. This reduced the transformation time by one week and eliminated the labor and materials required for one transfer. In studies evaluating different transformation vectors, we found hygromycin selection to be more efficient than BASTA for the production of transgenic plants. After co-cultivation, callus selected using BASTA required two subculture steps prior to regeneration, whereas higher transformation efficiencies were achieved from hygromycin-selected callus transferred to regeneration media after only one subculture. Our vector comparisons also demonstrated that the promoter driving the selectable marker greatly affected transformation efficiency with the maize ubiquitin promoter demonstrating the highest efficiency among the promoters tested. Using our optimized protocol we achieved an average transformation efficiency of 42%. Significantly, this high efficiency was achieved in a production setting where calluses were transferred and discarded on a set timetable to minimize labor and space required per transgenic line produced. These improvements provide considerable time and cost savings when transformations are conducted on a large scale.

Using our optimized transformation method, we generated 8,491 fertile T-DNA lines making the WRRC collection the largest collection of T-DNA lines in any grass with the exception of rice. To increase the utility of this collection, we used inverse PCR to sequence the DNA flanking the insertion sites. Our initial experiments focused on optimizing the IPCR method. We found 20–26% higher FST recovery when we performed sequencing reactions directly from the T-DNA into the genomic sequence compared to reactions from the genomic restriction site back toward the T-DNA. Adding sequencing reactions generated from a second enzyme digestion near the RB increased recovery of FSTs by approximately 25%, and we increased FST recovery by 20% when we included reactions from the LB. However, adding a third enzyme at the RB only produced a marginal increase in FST identification, and vectors with two LBs did not improve FST recovery.

**Figure 7:**
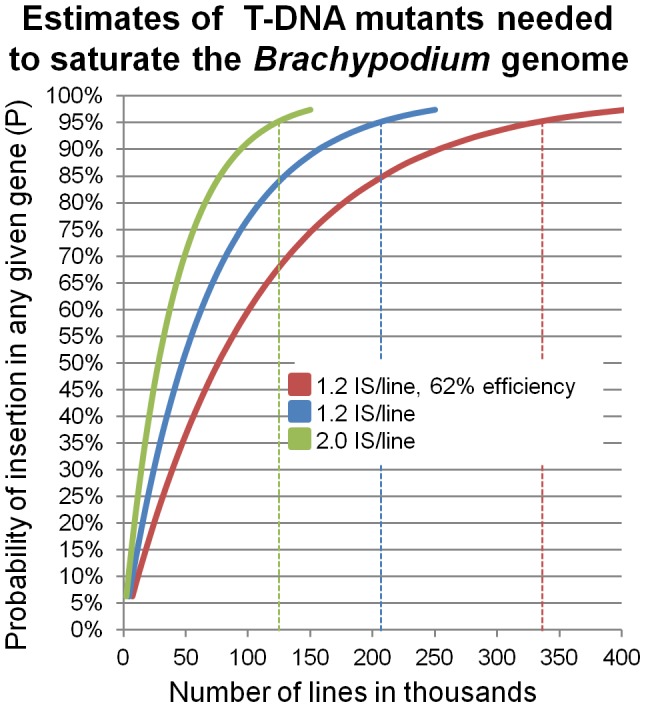
Estimated numbers of T-DNA mutants needed to saturate the *Brachypodium* genome. The number of T-DNA mutants needed for saturation mutagenesis of the *Brachypodium* genome is estimated using the formula {P = 1−(1−[x/g])^n^}. P is the probability of finding an insertion in a particular gene; g is the genome size in kb; x is the average gene length in kb; n is the number of T-DNA insertions needed. The red line shows the number of lines estimated using our current sequencing efficiency of identifying FSTs in 62% of the lines tested with an average of 1.2 IS/line. The blue and green lines illustrate estimates based on identifying FSTs in 100% of the lines with an average of 1.2 IS/line or 2.0 IS/line, respectively. Dashed lines indicate the number of lines needed for P = 0.95 for each efficiency of identifying insertion sites.

In total, we identified 5,285 specific insertion sites in the *Brachypodium* genome. We successfully identified T-DNA insertions in 62% of the lines that we sequenced and found an average of 1.2 insertions sites per line. These sites represent 1,499 insertions in genes and another 2,203 insertions within 1 kb of a gene. This latter class of insertions may alter gene expression if they lie within regulatory regions or if the T-DNA is an activation tagging construct. The WRRC collection comprises mutants with insertions in or near (within 1 kb) 8.8% (2,245) of the annotated *Brachypodium* genes and represents a significant addition to existing collections of available *Brachypodium* insertional mutants. However, the number of lines is far from saturation. The formula {P = 1−(1−[x/g])^n^} [61] can be used to calculate the probability (P) of finding an insertion in a particular gene (“g” is the genome size in kb, “x” is the average gene length in kb, and “n” is the number of T-DNA insertions needed). Applying the above formula to *Brachypodium* assuming our current 62% efficiency of retrieving a useful FST from a line and an average of 1.2 T-DNA insertions detected per line, we calculate that a collection of 76,000 lines would have a 50% probability of containing an insertion in any average 3.3 kb *Brachypodium* gene (**Fig. 7**). To reach P = 0.95, a collection on the order of 329,000 lines would be necessary. Due to redundancy of hits, the first T-DNA insertions will hit the highest diversity of genes. Thus, even the smaller P = 0.5 collection would have great utility. These are large, yet not unrealistic, numbers. This estimation assumes random integration, but because T-DNA insertions are preferentially identified near genes, fewer lines should be needed to reach saturation. Furthermore, the utility of the existing collections can be increased through improvements in detection of insertions missed in the first sequencing attempts (**Fig. 7**). Current efforts are focused on identifying insertion sites in the 38% of the lines of the WRRC collection lacking FSTs and increasing the average number of T-DNA insertions detected to approach the expected number of 2 per line (estimated at 9,000 additional insertion sites).

The insertion sites are accessible through the project website (http://brachypodium.pw.usda.gov/) where they can be searched by BLAST, Gbrowse or downloaded as a table. Lines are freely available to anyone in the scientific community. The WRRC T-DNA collection represents a significant and growing resource for plant science research.

## Supporting Information

Table S1 **Cloning primer sequences.**
(DOC)

Table S2 **Excel file containing a complete list of FST sequences with their genomic locations and sequencing information.**
(XLS)

File S1 **Text file containing a list of all FST sequences in FASTA format.**
(FASTA)

## References

[1] Catalán P, Müller J, Hasterok R, Jenkins G, Mur LAJ, et al (2012) Evolution and taxonomic split of the model grass *Brachypodium distachyon* . Annals of Botany Available:http://www.ncbi.nlm.nih.gov/pubmed/22213013. Accessed 26 January 2012. 10.1093/aob/mcr294 PMC3268539 22213013

[2] Draper J, Mur LA, Jenkins G, Ghosh-Biswas GC, Bablak P, et al (2001) *Brachypodium distachyon*. A new model system for functional genomics in grasses. Plant Physiol 127: 1539–1555. 11743099 PMC133562

[3] (2006) Breaking the Biological Barriers to Cellulosic Ethanol: A Joint Research Agenda, US. Department of Energy, Office of Science and Office of Energy Efficiency Available at: http://genomicsgtl.energy.gov/biofuels/.

[4] Carpita NC (1996) Structure and biogenesis of the cell walls of grasses. Annu Rev Plant Physiol Plant Mol Biol 47: 445–476 doi:10.1146/annurev.arplant.47.1.445. 15012297 10.1146/annurev.arplant.47.1.445

[5] Vogel J (2008) Unique aspects of the grass cell wall. Curr Opin Plant Biol 11: 301–307 doi:10.1016/j.pbi.2008.03.002. 18434239 10.1016/j.pbi.2008.03.002

[6] Bevan MW, Garvin DF, Vogel JP (2010) *Brachypodium distachyon* genomics for sustainable food and fuel production. Curr Opin Biotechnol 21: 211–217 doi:10.1016/j.copbio.2010.03.006. 20362425 10.1016/j.copbio.2010.03.006

[7] Garvin D, Gu Y, Hasterok R, Hazen SP, Jenkins G, et al (2008) Development of genetic and genomic research resources for *Brachypodium distachyon*, a new model system for grass crop research. The Plant Genome 48: 69–84.

[8] International *Brachypodium* Initiative (2010) Genome sequencing and analysis of the model grass *Brachypodium distachyon* . Nature 463: 763–768 doi:10.1038/nature08747. 20148030 10.1038/nature08747

[9] Brkljacic J, Grotewold E, Scholl R, Mockler T, Garvin DF, et al (2011) *Brachypodium* as a model for the grasses: today and the future. Plant Physiol 157: 3–13 doi:10.1104/pp.111.179531. 21771916 10.1104/pp.111.179531 PMC3165879

[10] Bragg J, Tyler L, Vogel JP (2010) *Brachypodium distachyon*, a model for bioenergy crops. Handbook of Bioenergy Crop Plants. CRC Press.

[11] Mur LAJ, Allainguillaume J, Catalán P, Hasterok R, Jenkins G, et al (2011) Exploiting the *Brachypodium* Tool Box in cereal and grass research. New Phytol 191: 334–347 doi:10.1111/j.1469-8137.2011.03748.x. 21623796 10.1111/j.1469-8137.2011.03748.x

[12] Vogel JP, Tuna M, Budak H, Huo N, Gu YQ, et al (2009) Development of SSR markers and analysis of diversity in Turkish populations of *Brachypodium distachyon* . BMC Plant Biol 9: 88 doi:10.1186/1471-2229-9-88. 19594938 10.1186/1471-2229-9-88 PMC2719641

[13] Wiebe K, Harris NS, Faris JD, Clarke JM, Knox RE, et al (2010) Targeted mapping of Cdu1, a major locus regulating grain cadmium concentration in durum wheat (*Triticum turgidum* L. var *durum*). Theor Appl Genet 121: 1047–1058 doi:10.1007/s00122-010-1370-1. 20559817 10.1007/s00122-010-1370-1

[14] Rosewarne GM, Singh RP, Huerta-Espino J, Herrera-Foessel SA, Forrest KL, et al (2012) Analysis of leaf and stripe rust severities reveals pathotype changes and multiple minor QTLs associated with resistance in an Avocet×Pastor wheat population. Theor Appl Genet 124: 1283–1294 doi: 10.1007/s00122-012-1786-x. 22274764 10.1007/s00122-012-1786-x

[15] Bouteillé M, Rolland G, Balsera C, Loudet O, Muller B (2012) Disentangling the intertwined genetic bases of root and shoot growth in Arabidopsis. PLoS ONE 7: e32319 doi:10.1371/journal.pone.0032319. 22384215 10.1371/journal.pone.0032319 PMC3286473

[16] Brown AN, Lauter N, Vera DL, McLaughlin-Large KA, Steele TM, et al (2011) QTL mapping and candidate gene analysis of telomere length control factors in maize (*Zea mays* L.). G3 (Bethesda) 1: 437–450 doi:10.1534/g3.111.000703. 22384354 10.1534/g3.111.000703 PMC3276162

[17] Liu L, Stein A, Wittkop B, Sarvari P, Li J, et al (2012) A knockout mutation in the lignin biosynthesis gene *CCR1* explains a major QTL for acid detergent lignin content in *Brassica napus* seeds. Theor Appl Genet 124: 1573–1586 doi: 10.1007/s00122-012-1811-0. 22350089 10.1007/s00122-012-1811-0

[18] Vogel J, Hill T (2008) High-efficiency *Agrobacterium*-mediated transformation of *Brachypodium distachyon* inbred line Bd21-3. Plant Cell Rep 27: 471–478 doi:10.1007/s00299-007-0472-y. 17999063 10.1007/s00299-007-0472-y

[19] Vain P, Worland B, Thole V, McKenzie N, Alves SC, et al (2008) *Agrobacterium*-mediated transformation of the temperate grass *Brachypodium distachyon* (genotype Bd21) for T-DNA insertional mutagenesis. Plant Biotechnol J 6: 236–245 doi:10.1111/j.1467-7652.2007.00308.x. 18004984 10.1111/j.1467-7652.2007.00308.x

[20] Huo N, Gu YQ, Lazo GR, Vogel JP, Coleman-Derr D, et al (2006) Construction and characterization of two BAC libraries from *Brachypodium distachyon*, a new model for grass genomics. Genome 49: 1099–1108 doi:10.1139/g06-087. 17110990 10.1139/g06-087

[21] Huo N, Lazo GR, Vogel JP, You FM, Ma Y, et al (2008) The nuclear genome of *Brachypodium distachyon*: analysis of BAC end sequences. Funct Integr Genomics 8: 135–147 doi:10.1007/s10142-007-0062-7. 17985162 10.1007/s10142-007-0062-7

[22] Hasterok R, Marasek A, Donnison IS, Armstead I, Thomas A, et al (2006) Alignment of the genomes of *Brachypodium distachyon* and temperate cereals and grasses using bacterial artificial chromosome landing with fluorescence in situ hybridization. Genetics 173: 349–362 doi:10.1534/genetics.105.049726. 16489232 10.1534/genetics.105.049726 PMC1461447

[23] Sonah H, Deshmukh RK, Sharma A, Singh VP, Gupta DK, et al (2011) Genome-wide distribution and organization of microsatellites in plants: an insight into marker development in *Brachypodium* . PLoS ONE 6: e21298 doi:10.1371/journal.pone.0021298. 21713003 10.1371/journal.pone.0021298 PMC3119692

[24] Garvin DF, McKenzie N, Vogel JP, Mockler TC, Blankenheim ZJ, et al (2010) An SSR-based genetic linkage map of the model grass *Brachypodium distachyon* . Genome 53: 1–13 doi:10.1139/g09-079. 20130744 10.1139/g09-079

[25] Gu YQ, Ma Y, Huo N, Vogel JP, You FM, et al (2009) A BAC-based physical map of *Brachypodium distachyon* and its comparative analysis with rice and wheat. BMC Genomics 10: 496 doi:10.1186/1471-2164-10-496. 19860896 10.1186/1471-2164-10-496 PMC2774330

[26] Huo N, Garvin DF, You FM, McMahon S, Luo M-C, et al (2011) Comparison of a high-density genetic linkage map to genome features in the model grass *Brachypodium distachyon* . Theor Appl Genet 123: 455–464 doi:10.1007/s00122-011-1598-4. 21597976 10.1007/s00122-011-1598-4

[27] Vogel J, Garvin D, Leong O, Hayden D (2006) *Agrobacterium*-mediated transformation and inbred line development in the model grass *Brachypodium distachyon* . Plant Cell Tissue Organ Culture 85: 199–211.

[28] Filiz E, Ozdemir BS, Budak F, Vogel JP, Tuna M, et al (2009) Molecular, morphological, and cytological analysis of diverse *Brachypodium distachyon* inbred lines. Genome 52: 876–890 doi:10.1139/g09-062. 19935911 10.1139/g09-062

[29] Higgins JA, Bailey PC, Laurie DA (2010) Comparative genomics of flowering time pathways using *Brachypodium distachyon* as a model for the temperate grasses. PLoS ONE 5: e10065 doi:10.1371/journal.pone.0010065. 20419097 10.1371/journal.pone.0010065 PMC2856676

[30] Cao S, Kumimoto RW, Siriwardana CL, Risinger JR, Holt BF 3rd (2011) Identification and characterization of NF-Y transcription factor families in the monocot model plant *Brachypodium distachyon* . PLoS ONE 6: e21805 doi:10.1371/journal.pone.0021805. 21738795 10.1371/journal.pone.0021805 PMC3128097

[31] Faricelli ME, Valárik M, Dubcovsky J (2010) Control of flowering time and spike development in cereals: the earliness per se *Eps-1* region in wheat, rice, and *Brachypodium* . Funct Integr Genomics 10: 293–306 doi:10.1007/s10142-009-0146-7. 19851796 10.1007/s10142-009-0146-7 PMC2862174

[32] Barrero JM, Jacobsen JV, Talbot MJ, White RG, Swain SM, et al (2012) Grain dormancy and light quality effects on germination in the model grass *Brachypodium distachyon* . New Phytol 193: 376–386 doi:10.1111/j.1469-8137.2011.03938.x. 22039925 10.1111/j.1469-8137.2011.03938.x

[33] Guillon F, Larré C, Petipas F, Berger A, Moussawi J, et al (2012) A comprehensive overview of grain development in *Brachypodium distachyon* variety Bd21. J Exp Bot 63: 739–755 doi:10.1093/jxb/err298. 22016425 10.1093/jxb/err298 PMC3254678

[34] Guillon F, Bouchet B, Jamme F, Robert P, Quéméner B, et al (2011) *Brachypodium distachyon* grain: characterization of endosperm cell walls. J Exp Bot 62: 1001–1015 doi:10.1093/jxb/erq332. 21062963 10.1093/jxb/erq332

[35] Yordem BK, Conte SS, Ma JF, Yokosho K, Vasques KA, et al (2011) *Brachypodium distachyon* as a new model system for understanding iron homeostasis in grasses: phylogenetic and expression analysis of Yellow Stripe-Like (YSL) transporters. Ann Bot 108: 821–833 doi:10.1093/aob/mcr200. 21831857 10.1093/aob/mcr200 PMC3177677

[36] Mochida K, Yoshida T, Sakurai T, Yamaguchi-Shinozaki K, Shinozaki K, et al (2011) In silico analysis of transcription factor repertoires and prediction of stress-responsive transcription factors from six major gramineae plants. DNA Res 18: 321–332 doi:10.1093/dnares/dsr019. 21729923 10.1093/dnares/dsr019 PMC3190953

[37] Christensen U, Alonso-Simon A, Scheller HV, Willats WGT, Harholt J (2010) Characterization of the primary cell walls of seedlings of *Brachypodium distachyon*-a potential model plant for temperate grasses. Phytochemistry 71: 62–69 doi:10.1016/j.phytochem.2009.09.019. 19828160 10.1016/j.phytochem.2009.09.019

[38] Peraldi A, Beccari G, Steed A, Nicholson P (2011) *Brachypodium distachyon*: a new pathosystem to study Fusarium head blight and other Fusarium diseases of wheat. BMC Plant Biol 11: 100 doi:10.1186/1471-2229-11-100. 21639892 10.1186/1471-2229-11-100 PMC3123626

[39] Berkman PJ, Skarshewski A, Manoli S, Lorenc MT, Stiller J, et al (2012) Sequencing wheat chromosome arm 7BS delimits the 7BS/4AL translocation and reveals homoeologous gene conservation. Theor Appl Genet 124: 423–432 doi:10.1007/s00122-011-1717-2. 22001910 10.1007/s00122-011-1717-2

[40] Massa AN, Wanjugi H, Deal KR, O'Brien K, You FM, et al (2011) Gene space dynamics during the evolution of *Aegilops tauschii*, *Brachypodium distachyon*, *Oryza sativa*, and *Sorghum bicolor* genomes. Mol Biol Evol 28: 2537–2547 doi:10.1093/molbev/msr080. 21470968 10.1093/molbev/msr080 PMC3163431

[41] Ryu C-H, You J-H, Kang H-G, Hur J, Kim Y-H, et al (2004) Generation of T-DNA tagging lines with a bidirectional gene trap vector and the establishment of an insertion-site database. Plant Mol Biol 54: 489–502 doi:10.1023/B:PLAN.0000038257.93381.05. 15316285 10.1023/B:PLAN.0000038257.93381.05

[42] Yu S-M, Ko S-S, Hong C-Y, Sun H-J, Hsing Y-I, et al (2007) Global functional analyses of rice promoters by genomics approaches. Plant Molecular Biology 65: 417–425 doi:10.1007/s11103-007-9232-1. 17922261 10.1007/s11103-007-9232-1

[43] Weigel D, Ahn JH, Blázquez MA, Borevitz JO, Christensen SK, et al (2000) Activation tagging in Arabidopsis. Plant Physiol 122: 1003–1014 doi:10.1104/pp.122.4.1003. 10759496 10.1104/pp.122.4.1003 PMC1539247

[44] Jeon J, Lee S, Jung K, Jun S, Jeong D, et al (2000) T-DNA insertional mutagenesis for functional genomics in rice. Plant Journal 22: 561–570. 10886776 10.1046/j.1365-313x.2000.00767.x

[45] Wan S, Wu J, Zhang Z, Sun X, Lv Y, et al (2008) Activation tagging, an efficient tool for functional analysis of the rice genome. Plant Molecular Biology 69: 69–80 doi:10.1007/s11103-008-9406-5. 18830797 10.1007/s11103-008-9406-5

[46] Bablak P, Draper J, Davey M, Lynch P (1995) Plant regeneration and micropropagation of *Brachypodium distachyon* . Plant Cell, Tissue and Organ Cult 42: 97–107.

[47] Christiansen P, Andersen CH, Didion T, Folling M, Nielsen KK (2005) A rapid and efficient transformation protocol for the grass *Brachypodium distachyon* . Plant Cell Rep 23: 751–758 doi:10.1007/s00299-004-0889-5. 15503032 10.1007/s00299-004-0889-5

[48] Thole V, Worland B, Wright J, Bevan MW, Vain P (2010) Distribution and characterization of more than 1000 T-DNA tags in the genome of *Brachypodium distachyon* community standard line Bd21. Plant Biotechnol J 8: 734–747 doi:10.1111/j.1467-7652.2010.00518.x. 20374523 10.1111/j.1467-7652.2010.00518.x

[49] Trijatmiko K (2005) Activation tagging using the En-I and Ac-Ds maize transposon systems in rice. In: Comparative analysis of drought resistance genes in Arabidopsis and rice. Wageningen, The Netherlands: Wageningen University.

[50] Kumar CS, Wing RA, Sundaresan V (2005) Efficient insertional mutagenesis in rice using the maize En/Spm elements. Plant J 44: 879–892 doi:10.1111/j.1365-313X.2005.02570.x. 16297077 10.1111/j.1365-313X.2005.02570.x

[51] Thole V, Worland B, Snape JW, Vain P (2007) The pCLEAN dual binary vector system for *Agrobacterium*-mediated plant transformation. Plant Physiol 145: 1211–1219 doi:10.1104/pp.107.108563. 17932303 10.1104/pp.107.108563 PMC2151721

[52] Christensen AH, Quail PH (1996) Ubiquitin promoter-based vectors for high-level expression of selectable and/or screenable marker genes in monocotyledonous plants. Transgenic Research 5: 213–218 doi:10.1007/BF01969712. 8673150 10.1007/BF01969712

[53] Podevin N, De Buck S, De Wilde C, Depicker A (2006) Insights into recognition of the T-DNA border repeats as termination sites for T-strand synthesis by *Agrobacterium tumefaciens* . Transgenic Res 15: 557–571 doi:10.1007/s11248-006-9003-9. 16830227 10.1007/s11248-006-9003-9

[54] Lazo GR, Stein PA, Ludwig RA (1991) A DNA transformation-competent Arabidopsis genomic library in *Agrobacterium* . Biotechnology (NY) 9: 963–967. 10.1038/nbt1091-963 1368724

[55] Mori M, Tomita C, Sugimoto K, Hasegawa M, Hayashi N, et al (2007) Isolation and molecular characterization of a Spotted leaf 18 mutant by modified activation-tagging in rice. Plant Mol Biol 63: 847–860 doi:10.1007/s11103-006-9130-y. 17273822 10.1007/s11103-006-9130-y

[56] Jeong D-H, An S, Kang H-G, Moon S, Han J-J, et al (2002) T-DNA insertional mutagenesis for activation tagging in rice. Plant Physiol 130: 1636–1644 doi:10.1104/pp.014357. 12481047 10.1104/pp.014357 PMC166679

[57] Jeong D-H, An S, Park S, Kang H-G, Park G-G, et al (2006) Generation of a flanking sequence-tag database for activation-tagging lines in japonica rice. Plant J 45: 123–132 doi:10.1111/j.1365-313X.2005.02610.x. 16367959 10.1111/j.1365-313X.2005.02610.x

[58] An S, Park S, Jeong D-H, Lee D-Y, Kang H-G, et al (2003) Generation and analysis of end sequence database for T-DNA tagging lines in rice. Plant Physiol 133: 2040–2047 doi:10.1104/pp.103.030478. 14630961 10.1104/pp.103.030478 PMC300755

[59] Zhang J, Guo D, Chang Y, You C, Li X, et al (2007) Non-random distribution of T-DNA insertions at various levels of the genome hierarchy as revealed by analyzing 13 804 T-DNA flanking sequences from an enhancer-trap mutant library. Plant J 49: 947–959 doi:10.1111/j.1365-313X.2006.03001.x. 17253985 10.1111/j.1365-313X.2006.03001.x

[60] Alonso JM, Stepanova AN, Leisse TJ, Kim CJ, Chen H, et al (2003) Genome-wide insertional mutagenesis of *Arabidopsis thaliana* . Science 301: 653–657 doi:10.1126/science.1086391. 12893945 10.1126/science.1086391

[61] Krysan PJ, Young JC, Sussman MR (1999) T-DNA as an insertional mutagen in Arabidopsis. Plant Cell 11: 2283–2290 doi:10.1105/tpc.11.12.2283. 10590158 10.1105/tpc.11.12.2283 PMC144136

