# Supplementary material for: Generation and Characterization of the Western Regional Research Center Brachypodium T-DNA Insertional Mutant Collection
Source: PLoS One. 2012 Sep 17;7(9):e41916. doi: 10.1371/journal.pone.0041916 (PMC3444500; doi:10.1371/journal.pone.0041916)
Supplement: Table S1 — Cloning primer sequences. (DOC) [file pone.0041916.s001.doc]

**Table S1.** Cloning primer sequences

| ***Primer name*** | Primer sequence |
| --- | --- |
| BAR BamHIF | ttGGATCCatgagcccagaacgacgc |
| BAR KpnIR | ttGGTACCtcagatctcggtgacggg |
| pL3-2LB-F1 | ATCTACCGCGGGCTTTACTAAGCT |
| pL3-2LB-R1 | TGTGTTATTAATTTGTCTAAGCGTCA |
| UbiHEF | ttGATCTTGATATCctgcagtgcagcgtg |
| UbiOLR | ggtgagttcaggctttttcatGGATCCtctagagtcgac |
| Hyg OLF | gtcgactctagaGGATCCatgaaaaagcctgaactcacc |
| Hyg EcoRIR | ttGAATTCctatttctttgccctcgg |
| 35S-Enh-f1 | AAGAATTCGATCCCCAACATGGTGG |
| 35S-Enh-r1 | GCCGAATTCTAGATATCACATCAATCCACTT |
| RB-f2 | TATGCATGCGTTAACCTAGGAAGCTTGGCGCGCCAATTAAACTATCAGTG |
| RB-r2 | GTTGGCATGCACATACAAATGGACGAAC |
| GFP-f2 | TTCTAAGCTTGTTAACCTAGGAGCTCGGCGCGCCAATTAAACTATCAGTG |
| GFP-r2 | ACGAGGcgCgccGTACAAGGTACAGACTT |
| Nos-f | ATTTCCCCGGGCGTTCAAACAT |
| Nos-r | TCAAACCCGGGTAGTTTAATTCCCGA |
